# Risk factors and mortality of carbapenem-resistant *Klebsiella pneumoniae* bloodstream infection in a tertiary-care hospital in China: an eight-year retrospective study

**DOI:** 10.1186/s13756-022-01204-w

**Published:** 2022-12-19

**Authors:** Jie Chen, Hua Ma, Xiaoming Huang, Yanhui Cui, Wenzhong Peng, Fei Zhu, Shiyang Ma, Minjun Rao, Peipei Zhang, Hang Yang, Longxiang Su, Ruichao Niu, Pinhua Pan

**Affiliations:** 1 Department of Respiratory Medicine, National Key Clinical Specialty, Branch of National Clinical Research Center for Respiratory Disease, Xiangya Hospital, Central South University, No. 28 Xiangya Road, Kai-Fu District, Changsha, 410008 Hunan People’s Republic of China; 2 Center of Respiratory Medicine, Xiangya Hospital, Central South University, Changsha, 410008 Hunan People’s Republic of China; 3 Clinical Research Center for Respiratory Diseases in Hunan Province, Changsha, 410008 Hunan People’s Republic of China; 4 Hunan Engineering Research Center for Intelligent Diagnosis and Treatment of Respiratory Disease, Changsha, 410008 Hunan People’s Republic of China; 5 National Clinical Research Center for Geriatric Disorders, Xiangya Hospital, Changsha, 410008 Hunan People’s Republic of China; 6 Department of Infectious Disease, People’s Hospital of Liuyang City, Liuyang, 410300 Hunan People’s Republic of China; 7 Department of Respiratory Medicine, Traditional Chinese Medicine Hospital of Leiyang City, Hengyang, Hunan People’s Republic of China; 8 Department of Critical Care Medicine, State Key Laboratory of Complex Severe and Rare Diseases, Peking Union Medical College Hospital, Chinese Academy of Medical Science and Peking Union Medical College, 1st Shuaifuyuan, Dongcheng District, Beijing, 100730 People’s Republic of China; 9 Department of Respiratory Medicine, The Second Affiliated Hospital of Xinjiang Medical University, Urumqi, Xinjiang Uygur Autonomous Region People’s Republic of China

**Keywords:** Carbapenem resistance, *Klebsiella pneumoniae*, Bloodstream infection, Risk factors, Mortality, Intensive care units

## Abstract

**Background:**

The prevalence of carbapenem-resistant *Klebsiella pneumoniae* bloodstream infection (CRKP-BSI) is increasing worldwide. CRKP-BSI is associated with high rates of morbidity and mortality due to limited antibiotic choices. Here, we aim to identify the prevalence and risk factors for infection and mortality of CRKP BSI.

**Methods:**

This was a retrospective study of the past data from January 1st, 2012 to December 31st, 2019 of adult patients with KP-BSI in Xiangya Hospital, China.

**Results:**

Among the 706 incidences included in this study, 27.4% of them (212/753) being CR-KP strains. The occurrence of CRKP-BSI was increased from 20.69 to 37.40% from 2012 to 2019. Hematologic malignancies and ICU acquired infection were identified to be substantial risk factors of carbapenem resistance. The overall 28-day mortality rates of CRKP-BSI patients was significantly higher than that of CSKP-BSI (*P* < 0.001). Logistic regression analysis identified severe sepsis or septic shock incidents, inadequate empirical antimicrobial therapy and corticosteroids use preceding infection onset as the independent predictors of 28-day mortality of CRKP-BSI patients. However, high dose carbapenem combination therapy was identified as anticipated factors of low 28-day mortality.

**Conclusion:**

The occurrence of CRKP-BSI was significantly increased during the study period. Hematologic malignancies and ICU acquired infection were associated with the development of CRKP BSI. Severe sepsis or septic shock incidents, inadequate empirical antimicrobial therapy and corticosteroids use preceding infection onset caused significant increase of mortality rates in CRKP-BSI patients. High dose carbapenem combination therapy was associated with better outcome.

## Introduction

*Klebsiella pneumoniae* (KP) is a gram-negative bacteria commonly causing nosocomial infections including pneumonia, bloodstream infections (BSIs), hepatic abscess and urinary tract infections [1–4]. Although KP is thought to be the second pathogen after *Escherichia coli* (*E. coli*), that is responsible for gram-negative BSIs in adult patients, recent studies showed the incidence of KP-BSIs had exceeded the incidence of *E. coli* BSIs in intensive care unit (ICU) patients [5, 6]. KP-BSI infections have contributed to high health-care costs and an increased mortality. The crude mortality rate of KP-BSIs patients was reported to span from 20 to 40% [6–9].

Carbapenems, a class of broad-spectrum beta-lactam antibiotic drugs for the treatment of many Gram-negative bacteria, have been recommended in first-line therapies for multidrug-resistant KP infections [7]. However, during the last decade, the prevalence of carbapenem-resistant *Klebsiella pneumoniae* (CRKP) is increasing worldwide, such as Israel [4], Europe [5] and some South American countries [6, 7, 10]. In China, according to the data from CHINET (an antimicrobial resistance surveillance network in China) surveillance, the resistance rates of KP to imipenem and meropenem increased from 3.0% and 2.9% in 2005 to 26.3 and 25% in 2018 respectively [11]. Additionally, several reports have shown that resistance to carbapenem is related to the increase of KP-BSIs patients’ mortality [12–14]. Therefore, the situation is serious for CRKP-BSI patients.

Due to high morbidity and mortality and lack of appropriate medical intervention, CRKP-BSI has become a great challenge to clinical practitioners [4, 15, 16]. Recognizing the risk factors contributed to the development and mortality of CRKP-BSI may provide the basis for implementing of control measures and therapeutic strategies to prevent the CRKP BSI infection. Thus far, many clinical reports have demonstrated the various risk factors for both development and fatal outcomes of CRKP-BSI infections. For example, the source of skin and soft tissue infection, ICU-acquired infection, central venous catheter, mechanical ventilation, and previous antibiotic exposure were demonstrated to be powerful risk factors leading to the onset of CRKP-BSI [4, 12, 17], while mechanical ventilation, septic shock, inadequate empirical antibiotic therapy were reported to be independent mortality predictors of CRKP-BSI [4, 12]. However, the conclusions of these studies were inconsistent.

In this study, we not only evaluate the prevalence of CRKP-BSI in ICU, but also identify the risk factors related to infection and mortality of carbapenem resistance KP-BSI ICU.

## Methods

### Study design, setting and patients

A previous cohort study on patients with KP-BSIs was conducted between 1 January 2012 and 31 December 2019 in Xiangya Hospital, a tertiary healthcare hospital in Changsha, Hunan Province, China. Having approximately 3,500 beds (including 138 beds in 7 ICUs), it is known to be one of the largest comprehensive hospitals in China. This study was approved by the Xiangya Hospital Ethics Committee.

The study comprised of patients aged ≥ 18 years, who had been admitted to the hospital with KP-BSI during the period. The cases were CRKP-BSI infected patients, and the controls were patients with CSKP-BSI. In this report, only the initial positive cultures of KP in bloodstream for each patient were included. Recurrent infections were excluded. Patients with incomplete medical records or polymicrobial BSIs were also excluded. All patients were identified by searching the integrated hospital information system (IHIS), laboratory information system (LIS) and imageology achieving system (RIS) of Xiangya Hospital.

### Data collection

Results of clinical and microbial characteristics were obtained from the medical records by two experienced respiratory medical doctors. The clinical data collected included: patient demographics (age, gender), comorbidities (congestive heart failure, cerebrovascular disease, chronic lung disease, hepatobiliary and pancreatic diseases, kidney diseases, hematologic malignancy, solid tumor, solid organ transplantation, diabetes mellitus, immune diseases), the ward where onset of BSI was identified, previous exposures (previous healthcare interventions, such as hospitalization, surgery, dialysis, endoscopy and mechanical ventilation; previous antibiotics exposures), therapeutic management (choice of antibiotic), and outcomes (span of hospital stay, and mortality at 28 days). In addition, patients general state at the onset of BSI underwent adequate assessment, such as severe sepsis or septic shock. The Charlson comorbidity index (CCI) was used to determine the comorbid conditions as previously described [18]. Acute Physiology and Chronic Health Evaluation II (APACHE II) score was used to calculate the severity of illness within 24 h following the onset of BSIs. Furthermore, the adequate empirical antimicrobial therapies described by Zarkotou were also taken into consideration [19].

### Definitions

KP-BSI was described to be a positive blood culture of CR-KP strain collected from a patient that showed symptoms and/or signs of the systemic inflammatory response syndrome. For patients that had several incidences of CRKP-BSI, an unusual event was defined as independent occurrence at least 30 days after the final positive blood culture [10]. The date when the blood culture was collected is defined as the onset of BSI. KP-BSIs were classified as healthcare-associated and community-acquired. Ward at the onset of BSI was defined as the first positive blood culture identified more than 2 days after the ward admission without a prior positive blood culture with the same pathogen in last 30 days. BSI sources were established based on the Centers for Disease Control and Prevention criteria [20]. BSI was considered as primary when no source was identified. Septic shock was defined as sepsis associated with organ dysfunction and persistent hypotension despite volume replacement. Combination therapy was defined as a regimen that includes two or more antibiotics, with at least one agent showing in vitro activity against the KP. An empirical antimicrobial therapy was described to be appropriate unless it included at least one drug displaying in vitro activity against the KP isolate, initiated within 48 h of the index blood culture, and given in adequate doses. High dose carbapenem combination therapy was only used for CRKP with meropenem MIC (≤ 16 mg/L). It was referred as combination of intravenous injection of 2 g meropenem every 8 h and infusion over 3 h.

### Microbiology

The Vitek 2 system (BioMérieux, Marcy 1′Étoile, France) was used in the clinical microbiology laboratory for isolate identification and antimicrobial susceptibility testing. KP isolates were considered as carbapenem-resistant KP (CRKP) isolates when they were resistant to one or more carbapenems tested in the clinical microbiology (i.e., ertapenem, imipenem, or meropenem).

### Statistical analysis

SPSS 20.0 (Chicago, IL, USA) was used for statistical analysis. Categorical variables were expressed as frequency counts and percentages with 95% confidence interval (95% CI). Continuous variables were expressed as median and interquartile ranges (IQRs). Pearson χ^2^ or Fisher’s exact tests were used to analyze categorical variables between groups, and Student’s *t*-test or the Mann–Whitney *U* test were used to compare continuous variables as appropriate. In analysis of risk factors for CRKP infection and mortality, univariable logistic regression analysis was performed. To identify the independent risk factors, a multivariate logistic regression model was generated to control the effects of confounding variables. Variables with *p*-value < 0.1 in univariate testing were incorporated into the model using a backward stepwise approach. A two-tailed *p* value of < 0.05 was considered statistically significant.

## Results

### Incidence and mortality of CRKP-BSIs over the past 8 years

During the 8-year study period, 706 events of KP-BSI were consecutively collected from 1 January 2012 and 31 December 2019 in Xiangya Hospital, and 30% (212/706) of these incidences were CRKP isolates. Over the past 8 years, the drug resistance rates of KP detected in blood samples to imipenem and meropenem has been increasing (details are shown in Table 1).

**Table 1:** Susceptibility of *Klebsiella pneumoniae* to antimicrobial agents from 2012 to 2019

Antimicrobial agents	2012 (n = 58)		2013 (n = 64)		2014 (n = 71)		2015 (n = 83)		2016 (n = 88)		2017 (n = 96)		2018 (n = 115)		2019 (n = 131)	
	R (%)	S (%)	R (%)	S (%)	R (%)	S (%)	R (%)	S (%)	R (%)	S (%)	R (%)	S (%)	R (%)	S (%)	R (%)	S (%)
Ampicillin	89.7	1.72	98.4	0	100.0	0	100.0	0	100.0	0	100.0	0	100.0	0	100.0	0
Ampicillin-sulbactam	43.1	53.4	43.8	56.2	43.7	56.3	45.8	54.2	47.7	52.3	47.9	52.1	50.4	49.6	52.7	47.3
Piperacillin-tazobactam	22.4	74.1	28.1	68.8	25.4	71.8	30.1	69.9	30.7	68.2	35.4	62.5	36.5	61.7	37.4	58.8
Cefoperazone-sulbactam	20.7	72.4	26.6	71.9	24.0	74.6	28.9	69.9	31.8	68.2	34.4	61.5	33.9	63.5	34.4	62.6
Cefazolin	53.4	46.6	45.3	54.7	52.1	47.9	49.4	50.6	51.1	48.9	53.1	46.9	52.2	47.8	53.4	46.6
Cefuroxime	44.8	55.2	46.9	53.1	47.9	52.1	47.0	53.0	47.7	52.3	47.9	52.1	49.6	50.4	51.1	48.9
Ceftazidime	27.6	72.4	26.6	71.9	26.8	73.2	31.3	68.7	31.8	68.2	35.4	61.5	35.7	64.3	38.2	61.8
Ceftriaxone	36.2	63.8	40.6	59.4	40.8	59.2	45.8	54.2	45.5	54.5	49.0	51.0	51.3	48.7	52.7	47.3
Cefepime	27.6	72.4	29.7	70.3	31.0	69.0	30.1	69.9	34.1	65.9	36.5	63.5	34.8	65.2	37.4	62.6
Cefotetan	19.0	77.6	25.0	75.0	22.5	77.5	27.7	72.3	33.0	67.0	34.4	65.6	37.4	62.6	38.9	61.1
Aztreonam	32.8	67.2	37.5	62.5	36.6	63.4	38.6	60.2	39.8	60.2	41.7	56.3	36.5	63.5	41.2	58.8
Ertapenem	19.0	81.0	21.9	78.1	22.5	77.5	27.7	72.3	30.6	69.3	32.3	67.7	33.9	66.1	36.6	63.4
Imipenem	20.7	79.3	20.3	79.7	22.5	77.5	26.5	73.5	30.6	69.3	30.2	69.8	33.0	67.0	35.9	64.1
Meropenem	20.7	79.3	21.9	78.1	22.5	77.5	27.7	72.3	29.5	70.5	31.3	68.7	33.9	66.1	36.6	63.4
Amikacin	11.1	87.9	12.5	87.5	16.9	83.1	19.3	79.5	19.3	80.7	20.8	79.2	22.6	77.4	24.4	75.6
Gentamicin	34.5	65.5	28.1	71.9	26.8	73.2	30.1	68.7	23.9	76.1	25.0	75.0	24.3	75.7	27.5	72.5
Tobramycin	20.7	69.0	18.8	76.6	15.5	80.3	22.9	77.1	22.7	75.0	21.9	68.8	19.1	70.4	24.4	68.7
Ciprofloxacin	31.0	58.6	35.9	62.5	40.8	56.3	38.6	61.4	37.5	60.2	38.5	59.4	35.7	60.9	40.5	54.2
Levofloxacin	29.3	62.1	28.1	71.9	25.4	74.6	26.5	72.3	31.8	67.0	33.3	66.7	33.9	63.5	35.1	63.4
SMZ-TMP	32.8	67.2	32.8	67.2	32.4	67.6	34.9	65.1	35.2	64.8	36.5	63.5	38.3	61.7	38.9	61.1
Tigecycline					0	98.9	1.0	97.9	2.6	95.7	3.1	95.4				

R Resistance, S susceptible, SMZ-TMP Trimethoprim-sulfamethoxazole

**Fig. 1:**
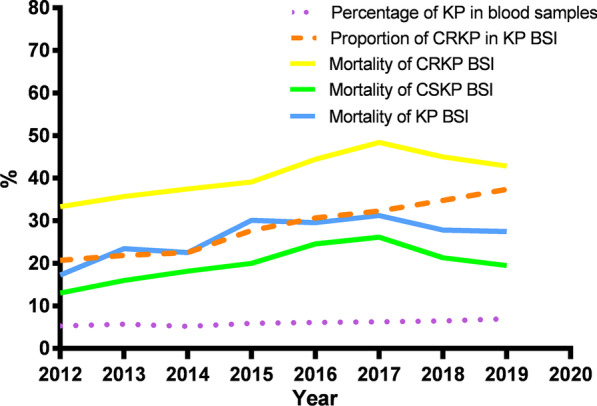
Trends in prevalence and mortality of *Klebsiella pneumoniae* bloodstream infections in Xiangya Hospital, Hunan Province, China, 2012–2019. KP: *Klebsiella pneumoniae*; CRKP: Carbapenem-resistant KP; CSKP: Carbapenem-susceptible KP; BSI: bloodstream infection

The percentage of KP in blood sample from 2012 to 2019 fluctuated from 5.3 to 7% during the 8 years (5.3 in 2012, 5.7 in 2013, 5.2 in 2014, 5.9 in 2015, 6.1 in 2016, 6.3 in 2017, 6.5 in 2018 and 7 in 2019). The percentage of CRKP in KP BSI from 2012 to 2019 were 20.69, 21.88, 22.54, 27.71, 30.68, 32.29, 34.78 and 37.40% respectively, which rose fastest from 2015 to 2019, as shown in Fig. 1. Additionally, the total 28-day mortality rate of KP-BSI patients was 26.1%, and it was higher in patients with CRKP-BSIs than in those with CSKP-BSIs (42.5% vs. 20.2%, respectively, *P* < 0.001) (Fig. 1). The mortality of KP BSI has increased from 17.24% in 2012 to 27.45% in 2019. The mortality of CRKP BSI and CSKP BSI increased from 33.33 and 13.04% in 2012 to 42.86 and 19.51% in 2019, respectively (Fig. 1).

### Clinical characteristics of patients

The average age of these 706 patients was 58.9 years (range: 18–92 years), and the male patients accounted for 66.9% (472/706). The most frequent comorbidities of these patients were kidney diseases (172/706, 24.4%), chronic lung diseases (146/706, 20.7%) and diabetes mellitus (140/706, 19.8%). The number of KP-BSI patients with the Carlson comorbidity index (CCI) ≥ 3 was 348 (49.3%). Most of the KP-BSIs cases were acquired at ICU (300/706, 42.5%) and most of them were undergoing hospitalization (378/706, 53.5%) within 12 months preceding infection onset. The most common KP infection source was primary bloodstream infections (408/706, 57.8%). Additionally, KP could be detected in samples from other sites (348/706, 49.3%), including pulmonary (166/706, 23.5%), pancreaticobiliary tract infection (40/706, 5.7%) and urinary tract infection (35/706, 5.0%), etc. 138 out of 706 (19.5%) KP-BSI patients (19.5%) were treated with corticosteroids and 150 out of 706 (21.2%) KP-BSI patients underwent chemotherapy or radiotherapy. 261 out of 706 (37.0%) KP-BSI patients did not receive adequate empirical antibiotic therapy. The number of KP-BSI patients with APACHE II score more than 15 was 240 (34.0%).

### Predictors of carbapenem-resistance among patients with Klebsiella BSI

We identified the clinical characteristics affecting the development of CRKP-BSIs, by comparing patient demographics, clinical characteristics, type of infections, and prior antibiotic exposures of patients with CRKP-BSIs and CSKP-BSIs (Table 2). The factors determined to be more relevantly related with CRKP-BSIs, using the univariable logistic regression analysis, included hematologic malignancy, ICU-acquired infection, undergoing hospitalization within 12 months preceding infection onset, undergoing surgery within 30 days preceding infection onset, inadequate empirical antibiotic therapy, corticosteroids use preceding infection onset and probable pulmonary source of infection. Using multivariate logistic regression analysis, hematologic malignancy (Odds ratio (OR) 4.68, 95% CI 2.3–9.4) and ICU-acquired infection (OR 2.10, 95% CI 1.3–3.4) was identified to be independent predictors of carbapenem-resistance among patients with Klebsiella BSI (Table 3).

**Table 2:** Comparison of clinical characteristics between patients with CRKP-BSI and CSKP-BSI

Variable	The total (N = 706)	CRKP (N = 212)	CSKP (N = 494)	*P* value
Male, n, (%)	472 (66.9)	138 (65.1)	334 (67.6)	0.515
Age(y), mean ± SD	58.9 ± 16.0	60.8 ± 16.0	58.1 ± 16.2	0.147
Comorbidities-No., %				
Congestive heart failure	96 (13.6)	22 (10.4)	74 (15.0)	0.102
Cerebrovascular disease	122 (17.3)	40 (18.9)	82 (16.6)	0.465
Chronic lung diseases	146 (20.7)	52 (24.5)	94 (19.0)	0.098
Hepatobiliary and pancreatic diseases	130 (18.4)	30 (14.2)	100 (20.2)	0.056
Kidney diseases	172 (24.4)	56 (26.4)	116 (23.5)	0.405
Hematologic malignancy	84 (11.9)	54 (25.5)	30 (6.1)	< 0.001
Solid tumor	118 (16.7)	40 (18.9)	78 (15.8)	0.315
Solid organ transplantation	49 (6.9)	19 (9.0)	30 (6.1)	0.166
Diabetes mellitus	140 (19.8)	36 (17.0)	104 (21.1)	0.214
Immune diseases	42 (5.9)	18 (8.5)	24 (4.9)	0.061
Charlson comorbidity index ≥ 3	348 (49.3)	112 (52.8)	236 (47.8)	0.218
Pre-infection healthcare interventions-No., %				
Enema^c^	52 (7.4)	18 (8.5)	34 (6.9)	0.453
Nasogastric catheter^b^	174 (24.6)	46 (21.7)	128 (25.9)	0.234
Urinary catheter^b^	254 (36.0)	82 (38.7)	172 (34.8)	0.327
Surgical drain^b^	104 (14.7)	38 (17.9)	66 (13.4)	0.117
Central venous catheter^b^	190 (26.9)	64 (30.2)	126 (25.5)	0.198
Peripheral arterial catheter^b^	65 (9.2)	26 (12.3)	39 (7.9)	0.066
Blood purification^b^	72 (10.2)	28 (13.2)	44 (8.9)	0.083
Tracheal cannula^b^	288 (40.8)	91 (42.9)	197 (39.9)	0.450
Tracheostomy^b^	54 (7.6)	20 (9.4)	34 (6.9)	0.242
Mechanical ventilation^c^	182 (25.8)	64 (30.2)	118 (23.9)	0.079
Gastroscopy^c^	15 (2.1)	6 (2.8)	9 (1.8)	0.394
Colonoscopy^c^	6 (0.8)	2 (0.9)	4 (0.8)	0.859
Bronchoscopy^c^	78 (11.0)	24 (11.3)	54 (10.9)	0.880
Sputum suction^b^	356 (50.4)	115 (54.2)	241 (48.8)	0.184
Thoracentesis^b^	56 (7.9)	21 (9.9)	35 (7.1)	0.204
Abdominocentesis^b^	35 (5.0)	12 (5.7)	23 (4.7)	0.573
Bone marrow puncture^b^	91 (12.9)	33 (15.6)	58 (11.7)	0.164
Lumbar puncture^b^	68 (9.6)	25 (11.8)	43 (8.7)	0.202
Previous surgery^b^	140 (19.8)	58 (27.4)	82 (16.6)	0.001
Parenteral nutrition^b^	237 (33.6)	77 (36.3)	160 (32.4)	0.311
Previous hospitalization^a^	378 (53.5)	134 (63.2)	244 (49.4)	0.001
Previous treatments administered^b^-No., %				
Corticosteroids	138 (19.5)	52 (24.5)	86 (17.4)	0.029
Chemotherapy or radiotherapy	150 (21.2)	40 (18.9)	110 (22.3)	0.311
Inadequate empirical antibiotic therapy	261 (37.0)	96 (45.3)	165 (33.4)	0.003
Previous use of antibiotics				
Carbapenems	218 (30.9)	68 (32.1)	150 (30.4)	0.652
Glycopeptides	144 (20.4)	48 (22.6)	96 (19.4)	0.332
Quinolones	227 (32.2)	74 (34.9)	153 (31.0)	0.305
3rd/4th generation cephalosporins	102 (14.4)	38 (17.9)	64 (13.0)	0.085
1st/2nd generation cephalosporins	86 (12.2)	25 (11.8)	61 (12.3)	0.836
Penicillins	35 (5.0)	8 (3.8)	27 (5.5)	0.342
β-lactamase inhibitor	308 (43.6)	101 (47.6)	207 (41.9)	0.159
Aminoglycosides	27 (3.8)	12 (5.7)	15 (3.0)	0.096
Linezolid	41 (5.8)	14 (6.6)	27 (5.5)	0.553
Tigecycline	77 (10.9)	28 (13.2)	49 (9.9)	0.199
Daptomycin	8 (1.1)	2 (0.9)	6 (1.2)	0.755
Nitroimidazoles	32 (4.5)	13 (6.1)	19 (3.8)	0.181
Source of BSI-No., %				
Primary	408 (57.8)	112 (52.8)	296 (59.9)	0.080
KP detection in samples from other sites	348 (49.3)	116 (54.7)	232 (47.0)	0.059
Pulmonary	166 (23.5)	66 (31.7)	100 (20.1)	0.001
Pleural effusion	16 (2.3)	6 (2.8)	10 (2.0)	0.510
Pancreaticobiliary tract infection	40 (5.7)	14 (6.6)	26 (5.3)	0.480
Live abscess	30 (4.2)	12 (5.7)	18 (3.6)	0.223
Urinary tract infection	35 (5.0)	11 (5.2)	24 (4.9)	0.853
Intestinal infection	26 (3.7)	6 (2.8)	20 (4.0)	0.431
Intra-abdominal infection	17 (2.4)	5 (2.4)	12 (2.4)	0.955
Skin infection	12 (1.7)	4 (1.9)	8 (1.6)	0.801
Cerebrospinal fluid	6 (0.8)	2 (0.9)	4 (0.8)	0.859
Ward at the onset of BSI-No., %				
Intensive care units	300 (42.5)	120 (56.6)	180 (36.4)	< 0.001
Medical wards	232 (32.9)	54 (25.5)	178 (36.0)	0.006
Surgical wards	170 (24.1)	38 (17.9)	132 (26.7)	0.012
Severity at BSI onset-No., %				
APACHE II score > 15	240 (34.0)	86 (40.6)	154 (31.2)	0.016
Severe sepsis/septic shock	244 (34.6)	88 (41.5)	156 (31.6)	0.011
Outcome-No., %				
28-day mortality	190 (26.9)	90 (42.5)	100 (20.2)	< 0.001

^a^During the 12 months preceding infection onset

^b^During the 30 days preceding infection onset

^c^During the 72 h preceding infection onset

**Table 3:** Multivariate analysis of factors leading to the development of CRKP-BSI

Variable	*P* value	OR	95% CI
Hematologic malignancy	< 0.001	4.68	2.322–9.427
Intensive care units acquired infection	0.003	2.101	1.291–3.420
Previous hospitalization^a^	0.761	1.097	0.603–1.996
Previous surgery^b^	0.247	1.464	0.767–2.794
Inadequate empirical antibiotic therapy^b^	0.150	1.485	0.866–2.546
Corticosteroids use preceding infection onset^b^	0.083	1.708	0.933–3.125
Pulmonary source of BSI	0.061	1.748	0.975–3.133

^a^During the 12 months preceding infection onset

^b^During the 30 days preceding infection onset

### Risk factors for mortality of CRKP-BSI patients

Overall, the 28-day mortality of CRKP-BSI patients was 42.5%. In order to identify the risk factors correlated with crude 28-day mortality of CRKP-BSI patients, we compared patient demographics, clinical characteristics, type of infections, and prior antibiotic exposures of deceased and survivor patients of CRKP-BSIs (Table 4). In the univariable logistic regression analysis, congestive heart failure and ICU-acquired infection were determined to be the main risk factors. Besides, the mortality rate was also affected by various events preceding CRKP-BSI onset, such as blood purification, mechanical ventilation, corticosteroids use preceding infection onset. Additionally, inadequate empirical antibiotic therapy, high APACHE II score, severe sepsis / septic shock were associated with the crude 28-day mortality in univariable logistic regression analysis, In contrast, mortality was lower in patients that underwent high doses of carbapenem combination therapy. In the final multivariable logistic regression analysis model, only corticosteroids use preceding infection onset (OR 6.45, 95% CI 1.12–37.08, *P* = 0.037), inadequate empirical antibiotic therapy (OR 15.01, 95% CI 3.70–60.79, *P* < 0.001), severe sepsis / septic shock (OR 8.44, 95% CI 1.84–38.39, *P* = 0.006) remained adversely and independently associated with 28-days mortality, whereas a protective effect was observed for high doses of carbapenem combination therapy (OR 0.11, 95% CI 0.03–0.51, *P* = 0.004) (Table 5).

**Table 4:** Univariate analysis of factors for 28-day mortality in patients with infections caused by CRKP-BSI

Variable	Death (N = 90)	Survivors (N = 122)	*P* value
Male, n, (%)	56 (62.2)	82 (67.2)	0.451
Age(y), mean ± SD	61.1 ± 17.7	60.7 ± 14.8	0.900
Comorbidities- No., %			
Congestive heart failure	14 (15.6)	8 (6.6)	0.034
Cerebrovascular disease	18 (20.0)	22 (18.0)	0.717
Chronic lung diseases	22 (24.4)	30 (24.6)	0.981
Hepatobiliary and pancreatic diseases	17 (18.9)	13 (10.7)	0.089
Kidney diseases	24 (26.7)	32 (26.2)	0.943
Hematologic malignancy	26 (28.9)	28 (23.0)	0.327
Solid tumor	14 (15.6)	26 (21.3)	0.290
Solid organ transplantation	9 (10.0)	10 (8.2)	0.650
Diabetes mellitus	12 (13.3)	24 (19.7)	0.224
Immune diseases	10 (11.1)	8 (6.6)	0.240
Charlson comorbidity index ≥ 3	46 (51.1)	66 (54.1)	0.667
Pre-infection healthcare interventions-No., %			
Enema^c^	9 (10.0)	9 (7.4)	0.498
Nasogastric catheter^b^	14 (15.6)	32 (26.2)	0.062
Urinary catheter^b^	34 (37.8)	48 (39.3)	0.817
Surgical drain^b^	16 (17.8)	22 (18.0)	0.962
Central venous catheter^b^	30 (33.3)	34 (27.9)	0.392
Peripheral arterial catheter^b^	15 (16.7)	11 (9.0)	0.093
Blood purification^b^	19 (21.1)	9 (7.4)	0.004
Tracheal cannula^b^	47 (48.9)	44 (38.5)	0.132
Tracheostomy^b^	11 (12.2)	9 (7.4)	0.233
Mechanical ventilation^c^	36 (40.0)	28 (23.0)	0.008
Gastroscopy^c^	3 (3.3)	3 (2.5)	0.704
Colonoscopy^c^	2 (2.2)	1 (0.8)	0.393
Bronchoscopy^c^	14 (15.6)	10 (8.2)	0.095
Sputum suction^b^	52 (57.8)	63 (51.6)	0.375
Thoracentesis^b^	11 (12.2)	10 (8.2)	0.332
Abdominocentesis^b^	6 (6.7)	6 (5.7)	0.586
Bone marrow puncture^b^	14 (15.6)	19 (15.6)	0.997
Lumbar puncture^b^	11 (12.2)	14 (11.5)	0.868
Previous surgery^b^	26 (28.9)	32 (26.2)	0.668
Parenteral nutrition^b^	37 (41.1)	40 (32.8)	0.213
Previous hospitalization^a^	60 (66.7)	74 (60.7)	0.370
Previous treatments administered^b^-No., %			
Corticosteroids	38 (42.2)	14 (11.5)	< 0.001
Chemotherapy or radiotherapy	12 (13.3)	28 (23.0)	0.077
Inadequate empirical antibiotic therapy	78 (86.7)	18 (14.8)	< 0.001
Previous use of antibiotics			
Carbapenems	30 (33.3)	38 (31.1)	0.736
Glycopeptides	22 (24.4)	26 (21.3)	0.590
Quinolones	34 (37.8)	40 (32.8)	0.451
3rd/4th generation cephalosporins	14 (15.6)	24 (19.7)	0.440
1st/2nd generation cephalosporins	10 (11.1)	15 (12.3)	0.792
Penicillin	4 (4.4)	4 (3.3)	0.660
β-lactamase inhibitor	49 (54.4)	52 (42.6)	0.088
Aminoglycosides	5 (5.6)	7 (5.7)	0.995
Linezolid	4 (4.4)	10 (8.2)	0.277
Tigecycline	13 (14.4)	15 (12.3)	0.648
Daptomycin	1 (1.1)	1 (0.8)	0.828
Nitroimidazoles	6 (6.7)	7 (5.7)	0.781
Source of BSI- No., %			
Primary	44 (48.9)	68 (55.7)	0.323
KP detection in samples from other sites	55 (61.1)	61 (50.0)	0.108
Pulmonary	20 (22.2)	36 (29.5)	0.234
Pleural effusion	2 (2.2)	4 (3.3)	0.647
Pancreaticobiliary tract infection	8 (8.9)	6 (4.9)	0.250
Live abscess	5 (5.6)	7 (5.7)	0.995
Urinary tract infection	7 (7.8)	4 (3.3)	0.144
Intestinal infection	4 (4.4)	2 (1.6)	0.223
Intra-abdominal infection	3 (3.3)	2 (1.6)	0.422
Skin infection	3 (3.3)	1 (0.8)	0.184
Cerebrospinal fluid	2 (2.2)	0 (3.3)	0.098
Ward at the onset of BSI- No., %			
Intensive care units	62 (68.9)	58 (47.5)	0.002
Medical wards	18 (20.0)	36 (29.5)	0.116
Surgical wards	10 (11.1)	28 (23.0)	0.026
Severity at BSI onset- No., %			
APACHE II score > 15	45 (50.0)	41 (33.6)	0.016
Severe sepsis/septic shock	60 (66.7)	28 (23.0)	< 0.001
Therapeutic management-No., %			
Monotherapy	7 (7.8)	20 (16.4)	0.063
Combination therapy	83 (93.3)	102 (83.6)	0.063
Combination with high doses of carbapenem	30 (33.3)	96 (78.7)	< 0.001
Tigecycline containing regimen	38 (42.2)	46 (37.7)	0.506
Aminoglycoside containing regimen	30 (33.3)	28 (23.0)	0.094
Polymyxin B containing regimen	12 (13.3)	16 (13.1)	0.963
Ceftazidime and avibactam containing regimen	10 (11.1)	8 (6.6)	0.240

^a^During the 12 months preceding infection onset

^b^During the 30 days preceding infection onset

^c^During the 72 h preceding infection onset

**Table 5:** Multivariate analysis of factors for 28-day mortality in patients with infections caused by CRKP-BSI

Variable	*P* value	OR	95% CI
Congestive heart failure	0.151	7.576	0.479–119.913
Intensive care units acquired infection	0.083	3.774	0.840–16.969
Blood purification^b^	0.384	2.893	0.265–31.623
Mechanical ventilation^c^	0.298	2.457	0.452–13.344
Corticosteroids use preceding infection onset^b^	0.037	6.451	1.122–37.081
Inadequate empirical antibiotic therapy	< 0.001	15.006	3.704–60.786
APACHE II score > 15	0.294	2.189	0.506–9.464
Severe sepsis/septic shock	0.006	8.435	1.854–38.385
Combination with high doses of carbapenem	0.004	0.114	0.026–0.508

^b^During the 30 days preceding infection onset

^c^During the 72 h preceding infection onset

## Discussion

CRKP-BSI, one of the global public health concerns, has gained much attention for its considerable mortality. In China, a rapid increase of CRKP isolates cases among all KP isolates has been reported since 2010, while the current national average reaches 15.6% [21]. It was also reported that the incidence of CRKP has ubiquitously increased all over the world [22, 23]. For high CRKP endemic areas such as United States, Greece, Israel, and Italy, the percentage of CRKP-BSI ranged between 18 and 68%. [24–27]. In the current study, we had an interesting observation of an increase in CRKP-BSI occurrence since 2012, which was significantly higher when comparing average occurrences in China. The possible reason may be attributed to differences in the investigated populations and the severity of the diseases.

To enhance the empirical therapy for CRKP-BSI and to control the emergence, we investigated the predictors of carbapenem-resistance among patients with Klebsiella BSI. Our results indicate that there are several predict factors involved in CRKP infection including hematologic malignancy, ICU acquired infection, hospitalization during the 12 months preceding infection onset, surgery during the 30 days preceding infection onset, inadequate empirical antibiotic therapy, corticosteroids use preceding infection onset and pulmonary source of BSI in univariate analyses. However, only two predict factors including hematologic malignancy and ICU acquired infection were demonstrated to be independently related to CRKP-BSI by multivariate analysis, which is partly consistent with other reports. [4, 7, 28]. For example, Tian et al. revealed that CRKP-BSIs corresponds to ICU acquired infection [4]. Zhang et al. demonstrated that hematologic malignancies were associated with the development of CRKP BSI [12]. Patients with hematologic malignancies usually have frequent long-term hospitalization, undergo more invasive procedures, are exposed with high-grade antibiotics, and have impaired immunological response, which may lead to the development of CRKP-BSI. Therefore, the reinforcement of hygiene protocols in healthcare facilities and rational use of antibiotics should be specially strengthened to prevent the development of CRKP-BSI for patients with hematologic malignancies.

Increasing antibiotic resistance in ICUs is a significant clinical challenge, and we revealed that 56.6% of CRKP isolates were collected from ICU patients, which was consistent with other reports [26, 29]. It indicates that prevention and control of the occurrence of CRKP-BSI should be focused on ICU. ICU has already been recognized as a factory of creating, disseminating, and amplifying antimicrobial resistance [30]. Nosocomial infection is easily obtained via the airborne and contact transmission of resistant bacteria in the confined environment of ICU [15]. Noteworthy, medical equipment and devices have been demonstrated to be common vectors of CRKP in hospitals, especially in ICUs [29]. The development and transmission of CRKP is much easier in ICU due to the heavy use of the medical equipment and devices for the invasive procedures. Moreover, most ICU patients have relatively serious complications and may be treated with broad spectrum antibiotics or with longer duration of antibiotics use, which may contribute to the induction of carbapenem resistance for KP. To reduce onset of CRKP infections, appropriate controls should be implemented after the admission in ICU, such as active surveillance culture, precautionary isolations, disinfection, initial fitting antibiotic therapy, and relevant antibiotic de-escalation [17, 31].

However, previous studies suggested other variables, including surgery within the preceding 90 days, severe chronic comorbidities, previous hospitalizations, indwelling central venous catheter, mechanical ventilation, a nasogastric tube, prior carbapenem administration, and recent exposure to antimicrobials [17, 32–36]. In our study, no antibiotic or invasive procedures were identified as risk factors for CRKP-BSI. This difference may arise from different definition of the infection of CRKP-BSI, the exposure durations to antibiotics, or different patients selected for this research.

In our report, the total mortality rate at 28th day after infection in patients with KP-BSI was 26.1%. Patients with CRKP-BSI had a significantly elevated mortality rate as compared that of CSKP-BSI (42.5 vs. 26.1, *p* < 0.001), which was consistent with some previous observation [7]. However, other studies contradicted our findings, which showed that the mortality rate between CRKP-BSI and CSKP-BSI was almost the same [17, 24]. The possible explanation for these conflicting results is that infection-related mortality was associated with several factors, such as host immunity, bacterial virulence, and the efficacy of antibiotics [17].

To further explore the risk factors involved in 28-day mortality, patient characteristics and the therapeutic interventions on CRKP-BSIs were extensively investigated in our study. After adjusting for numerous confounders, various parameters, such as corticosteroids use preceding infection onset, inadequate empirical antibiotic therapy, severe sepsis or septic shock and combination therapy with high doses of carbapenem were related to a higher crude 28-day mortality. In agreement with the result by Papadimitriou-Olivgeris et al., we found that the use of corticosteroids preceding infection onset might contribute to the deleterious outcomes of CRKP-BSI patients [37]. Corticosteroids may inhibit a broad range of immune responses, which may have negative effects on infection control and eventually lead to the acceleration of death. Additionally, the well-known association between septic shock and mortality of CR-KP BSI was also observed in our study [27, 38]. However, it is worth mentioning that corticosteroids have been used as adjuncts in the treatment of septic shock according to the guidelines proposed by the Surviving Sepsis Campaign [39]. Whether corticosteroids in the treatment of septic shock due to CP-KP effective or not still needs to be observed and should be validated through randomized controlled trials.

Due to the limited treatment options, inappropriate empirical antibiotic therapy was also demonstrated to be predictive factor for death in CRKP-BSI patients, which is consistent with other studies [38, 40]. Therefore, more attention should be taken to the initial appropriate antibiotic therapy for CRKP-BSI patients. The implementation of antimicrobial stewardship program and regular surveillance of resistance should be strengthened to avoid unnecessary antibiotic exposure.

Regarding the application of protective factors, we showed that there was a significant difference of the 28-day mortality rate between patients treated or not treated with high doses of carbapenem. Although Giannella et al. reported the lack of association between combination therapy with high doses of meropenem and the 14-day mortality, it remained as a protective factor in the multivariate model when adjusted the propensity score [41]. Moreover, they stratified their model according to meropenem MIC and found the benefit of high doses of meropenem therapy for CRKP with meropenem MIC ≥ 16 mg/L. In our study, patients received high dose carbapenem combination therapy only when they infected with CRKP of meropenem MIC ≤ 16 mg/L. Maybe it was easier to reach the pharmacokinetic/pharmacodynamic of the high-dose/prolonged-infusion regimens of meropenem for patients with lower meropenem MIC CRKP strains. Further studies need to be carried out to determine the effect of carbapenem MIC on outcome in patients treated with high dose carbapenem combination therapy. Additionally, in other studies, various risk factors associated with mortality of patients with CRKP-BSI were identified, including APACHE II score, liver failure, trachea cannula on the day of bacteremia [17], bedridden status, mechanical ventilation, hemodialysis [33], and Pitt bacteremia score [24]. This may be explained by selection bias of study population.

There are several limitations that should be mentioned in this study. Firstly, it was a retrospective analysis of patients from a single center. Clinical data was collected solely according to medical records instead of interviews and clinical examinations of patients with KP-BSI by equally trained doctors. Secondly, we only included cases of *K. pneumoniae* infection with positive blood cultures. However, cases that were suspected to have KP-BSI but did not have blood samples collected for culture were not included. Therefore, total number of reported KP-BSI incidences can be slightly lower than the actual one. Finally, the lack of more detailed microbiological data, therefore, data related to strain’s genotype was not available. According to the report by Zheng et al., the mechanisms of resistance of CRKP were related with the emergences of ESBL (extended spectrum beta-lactamase) and carbapenemase genes [42]. With respect to the ESBL genes, CTX-M type enzymes were reported as the most common type, while regarding the carbapenemase genes, KPC-2 was the most prevalent in China [42]. Further studies will be carried out on the molecular epidemiology of CRKP strains in our group.

## Conclusions

In conclusion, our results show that in our study hematologic malignancies and ICU-acquired infection were independent risk factors associated with the occurrence of CRKP-BSI. Septic shock, inadequate empirical antimicrobial therapy and corticosteroids use preceding infection onset caused significant increase of mortality rates in CRKP-BSI patients. Combination therapy with high dose carbapenem is associated with better outcome. These findings may serve as recommendations for treatments and prevention of CRKP-BSI patients in Changsha, Hunan Province, China.

## Abbreviations

APACHE II: Acute Physiology and Chronic Health Evaluation II
BSIs: Bloodstream infections
CCI: Charlson comorbidity index
CI: Confidence interval
COPD: Chronic obstructive pulmonary disease
CRKP: Carbapenem resistant *K. pneumoniae*
CRKP-BSI: Carbapenem-resistant *Klebsiella pneumoniae* bloodstream infection
*E. coli*: *Escherichia coli*
ESBL: Extended spectrum beta-lactamase
ICU: Intensive care unit
IQRs: Interquartile ranges
KP: *Klebsiella pneumoniae*
KP-BSI: *Klebsiella pneumoniae* bloodstream infection
OR: Odds ratio
